# Immunometabolic Status during the Peripartum Period Is Enhanced with Supplemental Zn, Mn, and Cu from Amino Acid Complexes and Co from Co Glucoheptonate

**DOI:** 10.1371/journal.pone.0155804

**Published:** 2016-05-31

**Authors:** Fernanda Batistel, Johan S. Osorio, Annarita Ferrari, Erminio Trevisi, Michael T. Socha, Juan J. Loor

**Affiliations:** 1 Department of Animal Sciences and Division of Nutritional Sciences, University of Illinois, 1207 West Gregory Drive, 61801, Urbana, IL, United States of America; 2 Istituto di Zootecnica Facoltà di Scienze Agrarie, Alimentari e Ambientali, Università Cattolica del Sacro Cuore, 29122, Piacenza, Italy; 3 Zinpro Corporation, 55344, Eden Prairie, MN, United States of America; University of Lleida, SPAIN

## Abstract

The peripartum (or transition) period is the most-critical phase in the productive life of lactating dairy cows and optimal supply of trace minerals through more bioavailable forms could minimize the negative effects associated with this phase. Twenty Holstein cows received a common prepartal diet and postpartal diet. Both diets were partially supplemented with an inorganic (INO) mix of Zn, Mn, and Cu to supply 35, 45, and 6 ppm, respectively, of the diet dry matter (DM). Cows were assigned to treatments in a randomized completed block design, receiving an daily oral bolus with INO or organic trace minerals (AAC) Zn, Mn, Cu, and Co to achieve 75, 65, 11, and 1 ppm supplemental, respectively, in the diet DM. Liver tissue and blood samples were collected throughout the experiment. The lower glutamic-oxaloacetic transaminase concentration after 15 days in milk in AAC cows indicate lower hepatic cell damage. The concentration of cholesterol and albumin increased, while IL-6 decreased over time in AAC cows compared with INO indicating a lower degree of inflammation and better liver function. Although the acute-phase protein ceruloplasmin tended to be lower in AAC cows and corresponded with the reduction in the inflammatory status, the tendency for greater serum amyloid A concentration in AAC indicated an inconsistent response on acute-phase proteins. Oxygen radical absorbance capacity increased over time in AAC cows. Furthermore, the concentrations of nitric oxide, nitrite, nitrate, and the ferric reducing ability of plasma decreased with AAC indicating a lower oxidative stress status. The expression of *IL10* and *ALB* in liver tissue was greater overall in AAC cows reinforcing the anti-inflammatory response detected in plasma. The greater overall expression of *PCK1* in AAC cows indicated a greater gluconeogenic capacity, and partly explained the greater milk production response over time. Overall, feeding organic trace minerals as complexed with amino acids during the transition period improved liver function and decreased inflammation and oxidative stress.

## Introduction

The transition period is the most-critical phase in the productive life of high-producing dairy cows and it is characterized by decreased liver function and increased inflammation and oxidative stress [1, 2]. Although inflammatory pathways play important roles in normal immune function helping the body adjust to and overcome infection with the purpose of restoring homeostasis, uncontrolled inflammatory conditions can be detrimental and reduce reproductive performance and milk production [3]. Therefore, the liver of early-lactating dairy cows that is under physiological stress is likely to have its functions more impaired due to inflammation.

Oxidative stress results when reactive forms of oxygen are produced faster than they can be neutralized by antioxidant physiologic mechanisms in tissues and blood. Increased production of free radicals and reactive oxygen species coupled with decreased antioxidant defense mechanisms leads to damage of macromolecules and deregulation of normal metabolism during oxidative stress [4, 5]. Evidence indicates that oxidative stress plays a key role in several pathological conditions connected with animal production, reproduction and welfare [6].

Trace minerals are essential for dairy cows because they are involved in several key biological processes such as gluconeogenesis (Co-containing methylmalonyl-CoA mutase), ureagenesis (Mn-containing Arginase), vitamin B12 synthesis, hemoglobin synthesis, and are essential for the activity of superoxide dismutases (Zn and Cu), catalases (Fe), and certain transcription regulators (Zn) [7]. Minerals are commonly supplemented to cattle in the form of inorganic salts (**INO**), preferably as sulfates. However, in ruminants, association of minerals with fiber fractions in feedstuffs and binding of minerals to undigested fiber constituents in the gastrointestinal tract may reduce bioavailability leading to deficiency of some trace minerals [8]. Trace mineral deficiency may depress immunity in transition cows [9]. As a result, the development of organic forms of trace minerals, such as minerals complexed with amino acids is an alternative to minimize the risk of mineral antagonism and enhance absorption efficiency [10]. Previous studies observed that organic trace minerals improved immune response in dairy cows [11] and calves [12], and also improved milk yield and reproductive performance [7].

The general hypothesis of the present study was that organic trace mineral (**AAC**) supplementation would help high-producing dairy cows achieve greater milk production and dry matter intake (**DMI**) by overcoming the immune, metabolic and physiologic stress of the peripartal period at least in part by altering liver function, inflammation, oxidative stress, and metabolism. In order to address this hypothesis we evaluated blood biomarkers and liver mRNA expression of genes related with liver function, inflammation, oxidative stress and metabolism. Data on milk production, body condition score (**BCS**), DMI, and milk composition have been reported elsewhere [13].

## Materials and Methods

### Animals, Experimental Design, and Dietary Treatments

The Institutional Animal Care and Use Committee (IACUC) of the University of Illinois approved all protocols for this study (protocol no. 12097). Details of the experiment design have been published previously [13]. Briefly, 20 multiparous Holstein cows were offered a common diet supplemented entirely with inorganic trace minerals to meet the National Research Council [14] requirements from -110 to -30 d prior calving. From -30 d to calving cows received a common prepartal diet, and from calving to 30 DIM a common postpartal diet. Both diets were partially supplemented with an INO mix of Zn, Mn, and Cu to supply 35, 45, and 6 ppm, respectively, of the total diet DM. At -30 d relative to parturition, cows were assigned to the treatments in a randomized complete block design, receiving an oral bolus with a mix of INO (n = 11) or AAC (n = 9) containing Zn, Mn, Cu, and Co to achieve 75, 65, 11, and 1 ppm supplemental, respectively, in the total diet DM. Inorganic trace minerals were provided in sulfate form and AAC were supplied via Availa^®^Zn, Availa^®^Mn, Availa^®^Cu, and COPRO^®^ (Zinpro Corporation, Eden Prairie, MN).

### Blood Samples and Biomarker Analyses

Blood was sampled from the coccygeal vein every Monday and Thursday before the morning feeding from d -30 to 30 relative to parturition. Samples were collected into evacuated serum tubes (BD Vacutainer, BD and Co., Franklin Lakes, NJ) containing either clot activator or lithium heparin for serum and plasma, respectively. After blood collection, tubes with lithium heparin were placed on ice and tubes with clot activator were kept at 4°C until centrifugation (~30 min). Serum and plasma were obtained by centrifugation at 1,900 × *g* for 15 min. Aliquots of serum and plasma were frozen (-80°C) until further analysis.

Blood samples were analyzed for several biomarkers of liver function [e.g. bilirubin, cholesterol, glutamic-oxaloacetic transaminase (**GOT**), and paraoxonase (**PON**)], inflammation [e.g. albumin, ceruloplasmin, serum amyloid A (**SAA**), haptoglobin, retinol, tocopherol and β-carotene] and oxidative stress [e.g. myeloperoxidase (**MPO**), nitric oxide (**NOx**), nitrite (**NO**_**2**_^**−**^), nitrate (**NO**_**3**_^**−**^), ferric reducing antioxidant power (**FRAP**)]. Albumin (catalog no. 0018250040), cholesterol (catalog no. 0018250540), bilirubin (catalog no. 0018254640), and GOT (catalog no. 0018257540) were determined using the IL Test purchased from Instrumentation Laboratory Spa (Werfen Co., Milan, Italy) in the ILAB 600 clinical auto-analyzer (Instrumentation Laboratory, Lexington, MA). Coefficients of variation within and between assays were in all cases lower than 10%. Haptoglobin was analyzed using the method described by Skinner et al. [15], while ceruloplasmin was assessed following the method proposed by Sunderman and Nomoto [16] but the acetate buffer was changed to 0.8 *M*, pH 6.4, and contained 0.31% Na-EDTA. The SAA concentration was determined with a commercial ELISA immunoassay kit (Tridelta Development Ltd., Maynooth, Co. Kildare, Ireland). Antioxidant potential was assessed as FRAP using the colorimetric method of Benzie and Strain [17]. The concentrations of NOx, NO_2_^−^, NO_3_^−^, and PON were determined exactly as described by (Trevisi et al., 2013). The concentrations of MPO, retinol, tocopherol [1] and β-carotene [18] were determined exactly as described previously. Bovine IL-6 plasma concentration was determined by a colorimetric sandwich ELISA using Bovine IL-6 Screening Set (#ESS0029 Endogen, Pierce, Rockford, IL) validated for use with plasma [19].

### Liver Biopsy, RNA Extraction, Primer Design and Evaluation, and Quantitative PCR

Liver was sampled via puncture biopsy [20] from cows under local anesthesia (i.m. 10 mL lidocaine HCl, 2% soln) at approximately 0800 h on -30, -15, 10, and 30 d relative to parturition. Liver was frozen immediately in liquid nitrogen and stored until further analysis. Specific details of RNA extraction from liver, cDNA synthesis, and quantitative reverse transcription PCR are presented in the supplementary material (**S1 File**). Briefly, RNA was extracted from samples using Qiazol reagent in combination with the miRNeasy® Mini Kit (Cat. #217004, Qiagen). The quality of RNA evaluated by RNA integrity number in the 2100 Bioanalyzer (Agilent Technologies Inc., Santa Clara, CA) was 7.2 ± 1.1. Twenty-three target genes involved in inflammation, oxidative stress, fatty acid metabolism, hepatokines, gluconeogenesis, and the methionine cycle were selected. Primers were designed via Primer Express 3.0.1 software (Applied Biosystems).

Quantitative PCR (qPCR) was performed in the ABI Prism 7900 HT SDS instrument (Applied Biosystems). Details of primer sequences, amplicon size, and sequencing results are presented in Tables A and B in S1 File.

### Statistical Analysis

Data were analyzed using the MIXED procedure of SAS 9.3 (SAS Institute Inc., Cary, NC) according to the following model:

$$Y_{ijklm}=\;µ+D_{i}+P_{j}+\;{DP}_{ij}+\;B_{k}+C_{ijkl}+\;T_{m}+DT_{im}+\;DPT_{ijm}+\;e_{ijklm}$$

Where *Y*_*ijklm*_ is the dependent variable; *μ* is the overall mean; *D*_*i*_ is the fixed effect of diet (i = 1, 2, 3); *Pj* is the fixed effect of parity (j = 1, 2, 3); *DP*_*ij*_ is the fixed effect of diet by parity interaction; *B*_*k*_ is the random effect of block (k = 1, …9); *C*_*ijkl*_ is the random effect of cow within treatment, parity, and block (l = 1,…., *n*_*ijk*_); *T*_*m*_ is the fixed effect of time (d or wk) of the experiment (m = 1,…,n); *DT*_*im*_ is the fixed effect of treatment by time interaction; *DPT*_*ijm*_ is the fixed effect of treatment, by parity, by time interaction; and *e*_*ijklm*_ is the residual error. The impact of cows removed from the experiment [13] was categorically (i.e., removed vs. survived) evaluated using Fisher’s exact test (PROC FREQ). Blood biomarkers analyzed at various time points were not equally spaced therefore an exponential correlation covariance structure SP (POW) was used for repeated measures. Blood biomarkers and gene expression results were log_2_-scale transformed if needed to comply with normal distribution of residuals. Statistical differences were declared significant at *P* ≤ 0.05. The individual data for each of the items reported in this manuscript are freely available at http://figshare.com (https://dx.doi.org/10.6084/m9.figshare.3362668.v1).

## Results

### Biomarkers

Per IACUC guidelines, a total of 5 cows in AAC and 1 cow in INO were removed from the experiment within the first week postpartum due to incidence of ketosis, retained fetal membranes, or abomasal displacement. An additional cow in AAC was removed due to a chronic case of *Staphylococcus aureus* mastitis. Data from these cows was not included in any statistical analysis. The Fisher’s analysis of the 5 cows in AAC and 1 cow in INO revealed that treatment had no impact (*P* = 0.10) on the numbers of cows that had to be removed after parturition due to the onset of a clinical disease [13].

#### Liver function

**Fig 1:**
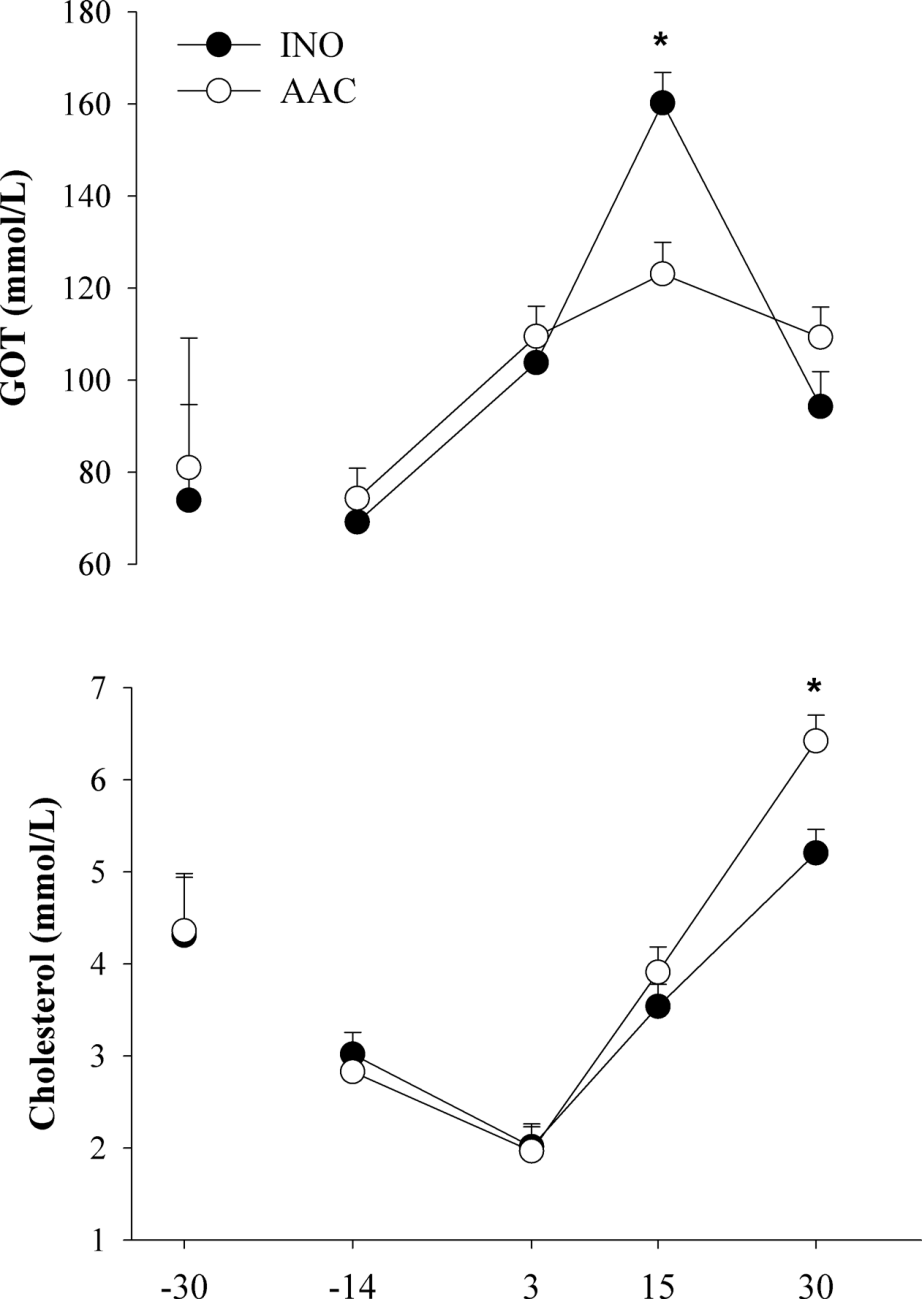
Effects of supplementing cows with inorganic (INO; n = 11) or organic (AAC; n = 9) trace minerals during the pre and postpartal periods on blood biomarkers of liver function. Mean separations between diets at a given time point were evaluated when a D × T effect (*P* ≤ 0.10) was observed. Differences (*) were declared at *P* ≤ 0.05.

Main effects of diet (D), time (T), and the interaction are presented in **Table 1**and **Fig 1**. A D × T was detected (*P* < 0.03) for GOT and cholesterol. The D × T in GOT occurred due to lower (*P* < 0.01) concentration at 15 d in AAC cows compared with INO. The D × T in cholesterol was a result of the greater concentration at 30 d (*P* < 0.01) in AAC compared with INO cows. The concentrations of bilirubin and PON were only affected by time (*P* < 0.01).

**Table 1:** Effects of supplementing cows with inorganic (INO) or organic (AAC) trace minerals during the peripartal period on blood biomarkers of liver function, inflammation and oxidative stress.

Parameter	Diets		SEM^1^	*P* value			
	INO	AAC		Diet	Parity^2^	Time	D×T^3^
Liver function							
Bilirubin, μmol/L^4^	2.83 (1.04)	2.39 (0.87)	0.30	0.28	—	<0.01	0.62
GOT, U/L	106.8	104.0	4.59	0.67	0.08	<0.01	<0.01
Cholesterol, mmol/L	3.44	3.78	0.20	0.22	—	<0.01	0.03
PON, U/mL	88.8	91.1	3.05	0.57	—	<0.01	0.38
Inflammation							
Albumin, g/L	34.9	35.9	0.54	0.17	0.02	0.34	0.04
Ceruloplasmin, μmol/L	3.84	3.46	0.15	0.10	—	< 0.01	0.27
SAA, μmol/mL	3.86	4.29	0.16	0.08	—	<0.01	0.72
Haptoglobin, g/L^4^	2.18 (-0.78)	2.39 (-0.87)	0.08	0.65	—	<0.01	0.09
IL-6, pg/mL	548.7	453.4	89.7	0.46	—	<0.01	0.01
Retinol, μg/100 mL^4^	22.65 (3.12)	30.88 (3.43)	0.73	0.05	—	<0.01	0.53
Tocopherol, μg/100 mL^4^	3.74 (1.32)	4.18 (1.43)	0.20	0.32	—	<0.01	0.60
β-carotene, μg/100 mL^4^	0.13 (-2.02)	0.14 (-1.97)	0.01	0.80	—	<0.01	0.60
Oxidative stress							
MPO, U/L	451.5	380.1	32.6	0.13	—	0.02	0.89
NO_x_, μmol/L	12.3	11.2	0.39	0.04	—	<0.01	0.69
NO_2_, μmol/L	6.2	5.4	0.25	0.03	—	<0.01	0.68
NO_3_, μmol/L	7.05	5.56	0.28	<0.01	—	0.12	0.91
FRAP, μmol/L	138.6	126.1	4.85	0.09	0.03	0.01	0.82

^1^Largest standard error of the mean is shown.

^2^Parity effect was used in the model depending on significance (*P* ≤ 0.15).

^3^Interaction of diet × time.

^4^ The values reported in parenthesis are the log-transformed data used for statistical analysis.

#### Inflammation

**Fig 2:**
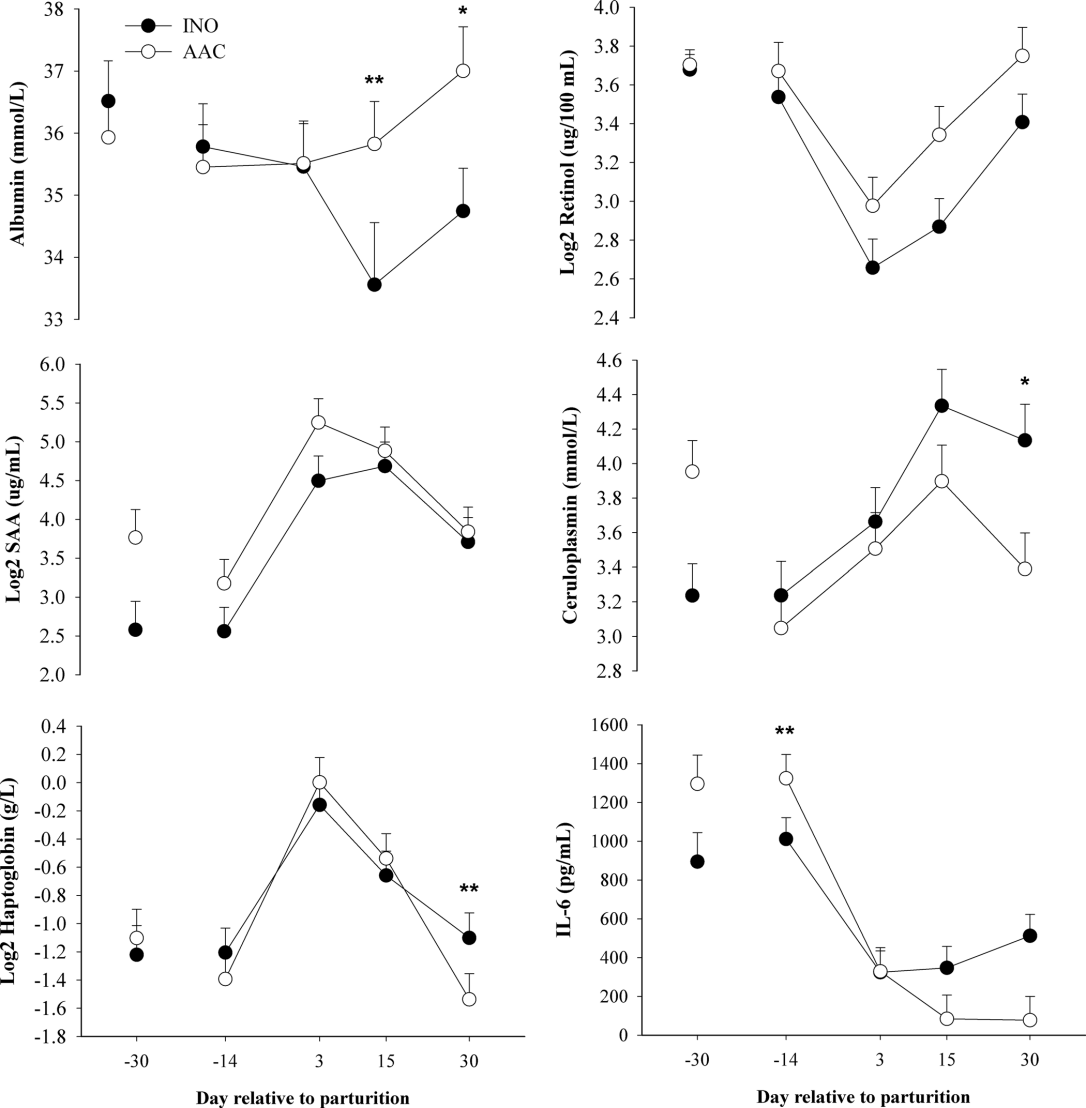
Effects of supplementing cows with inorganic (INO; n = 11) or organic (AAC; n = 9) trace minerals during the pre and postpartal periods on blood biomarkers of inflammation. Mean separations between diets at a given time point were evaluated when a D × T effect (*P* ≤ 0.10) was observed. Differences (*) were declared at *P* ≤ 0.05 and trends (**) at *P* ≤ 0.10.

Among the biomarkers related with inflammation, the concentration of albumin and IL-6 had a D × T (*P* < 0.04) (**Table 1**and **Fig 2**). Concentration of albumin tended to be greater at 15 (*P* = 0.07) and was greater at 30 d (*P* = 0.03) postpartum in AAC cows compared with INO. The D × T in IL-6 was associated with a trend (*P* = 0.07) for greater concentration at -14 d in AAC compared with INO cows, followed by lower (*P* = 0.01) postpartal concentration at 30 d in AAC compared with INO cows. There was a tendency (*P* < 0.10) for an overall treatment effect for the concentration of SAA, where AAC cows tended to have greater (*P* = 0.08) SAA in comparison with INO. Concentration of ceruloplasmin did not differ (*P* = 0.10).

A tendency for a D × T was detected for haptoglobin due to a greater (*P* = 0.09) concentration at d 30 in INO compared with AAC cows. Retinol concentration was overall greater (*P* = 0.05) in AAC compared with INO cows. There was no effect of treatment (*P* > 0.10) on the concentration of tocopherol and β-carotene; however, both were affected by time (*P* < 0.01). Tocopherol increased after parturition, whereas β-carotene decreased during the same time-frame.

#### Oxidative stress

**Fig 3:**
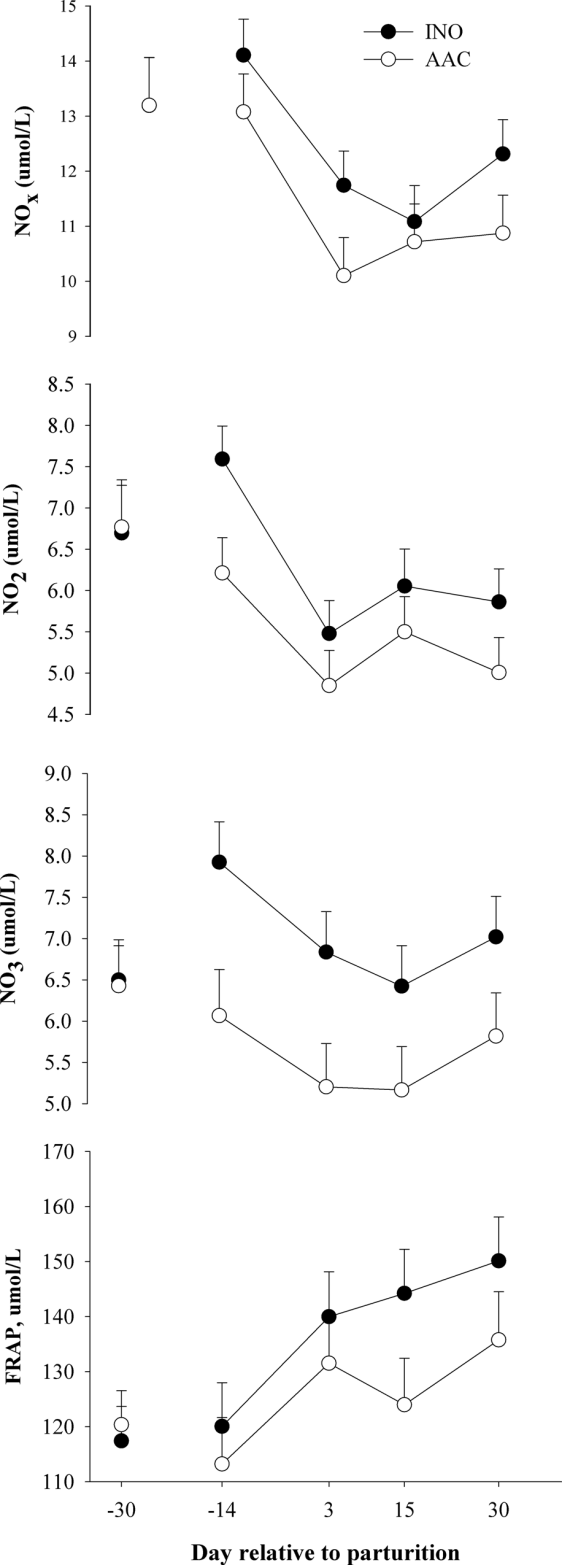
Effects of supplementing cows with inorganic (INO; n = 11) or organic (AAC; n = 9) trace minerals during the pre and postpartal periods on blood biomarkers of oxidative stress. Mean separations between diets at a given time point were evaluated when a D × T effect (*P* ≤ 0.10) was observed. Differences (*) were declared at *P* ≤ 0.05 and trends (**) at *P* ≤ 0.10.

The concentrations of NO_X_ (**Table 1**and **Fig 3**; *P* = 0.04), NO_2_ (*P* = 0.03), and NO_3_ (*P* < 0.01) were lower in AAC compared with INO cows and FRAP (*P* = 0.09) tended to be lower in the same group of cows. The concentration of MPO decreased over time (*P* = 0.02), with no treatment effect (*P* > 0.10).

### Target gene expression in liver tissue

#### Inflammation

**Fig 4:**
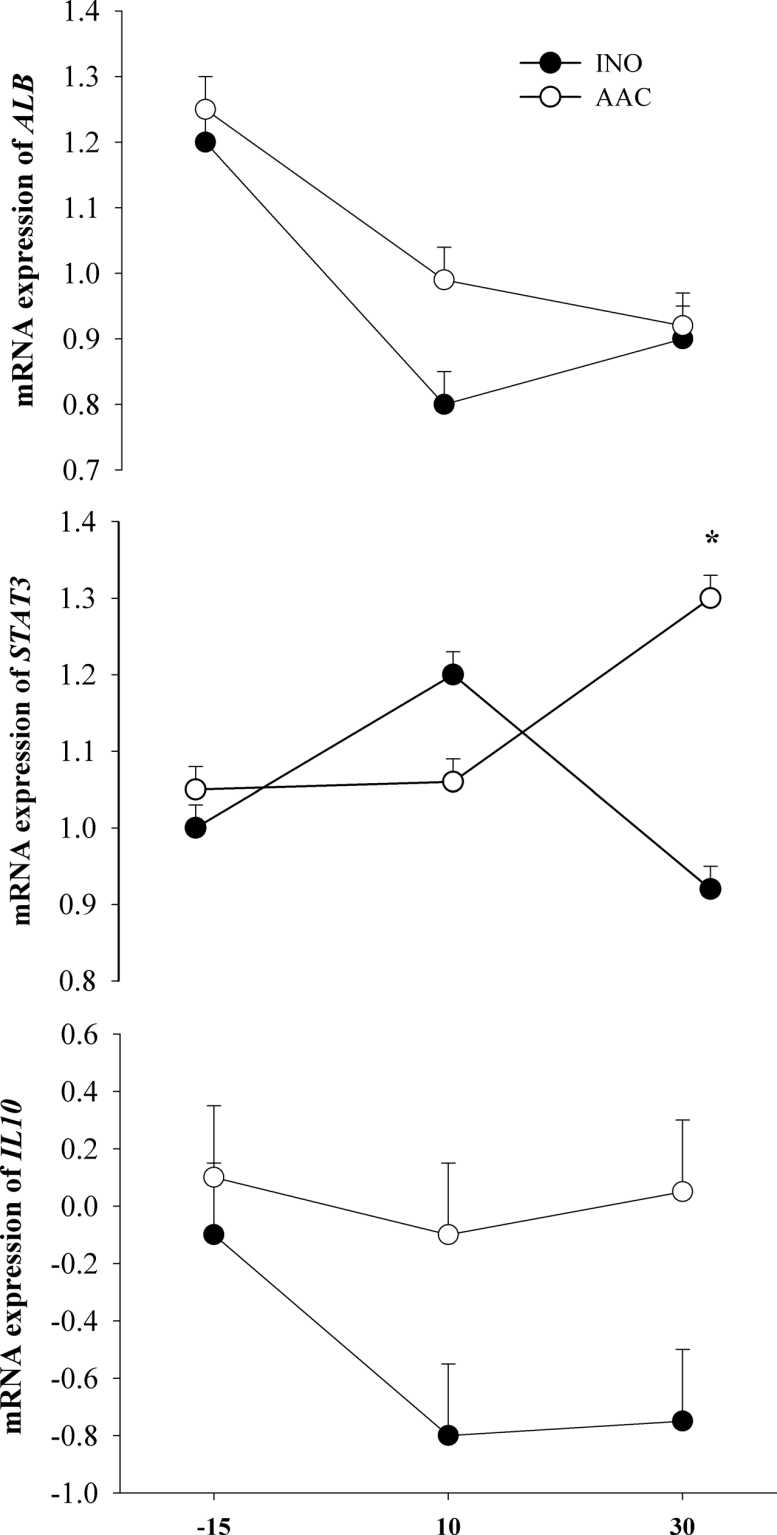
Effects of supplementing cows with inorganic (INO; n = 11) or organic (AAC; n = 9) trace minerals during the pre and postpartal periods on hepatic mRNA expression of genes related with inflammation. Mean separations between diets at a given time point were evaluated when a D × T effect (*P* ≤ 0.10) was observed. Differences (*) were declared at *P* ≤ 0.05 and trends (**) at *P* ≤ 0.10.

Among the chosen genes related with inflammatory response, only the mRNA expression of the transcription factor *STAT3* had a D × T interaction (*P* < 0.01) due to an increase in expression over time, primarily at 30 d (*P* < 0.01) postpartum, in response to AAC (**Table 2**and **Fig 4**). Similarly, there was an overall treatment effect for greater expression of the anti-inflammatory cytokine *IL10* (*P* = 0.03) and the negative acute-phase protein *ALB* (*P* < 0.01) in cows fed AAC in comparison with INO. The expression of the transcription factor *NFKB1* as well as the positive acute-phase protein (**APP**) gene *HP* decreased over time (*P* < 0.01).

**Table 2:** Effects of supplementing cows with inorganic (INO) or organic (AAC) trace minerals during the peripartal period on expression of genes related with inflammation response, oxidative stress, fatty acid metabolism, hepatokines and the methionine cycle.

Gene^1^	Diets		SEM^2^	*P* value		
	INO	AAC		Diet	Time	D×T^3^
Inflammation response						
*ALB*	1.02	1.11	0.01	<0.01	<0.01	0.53
*STAT3*	1.05	1.14	0.04	0.13	0.14	<0.01
*NFKB1*	0.78	0.85	0.06	0.36	0.05	0.74
*TNF*	0.73	0.84	0.06	0.22	0.23	0.35
*SAA2*	-0.69	-0.59	0.35	0.84	0.11	0.71
*CP*	1.09	0.97	0.11	0.44	0.65	0.34
*HP*	-2.02	-1.85	0.50	0.80	0.02	0.56
*IL1B*	-0.91	-0.50	0.19	0.14	0.12	0.97
*IL10*	-0.52	-0.01	0.16	0.03	0.16	0.57
Oxidative stress						
*SOD1*	0.98	0.95	0.03	0.55	0.60	0.11
*SOD2*	9.96	10.43	0.58	0.56	0.77	0.04
*PON1*	1.03	1.22	0.08	0.13	<0.01	0.41
*NOS2*	-0.12	-0.25	0.12	0.41	<0.01	0.46
Fatty acid metabolism						
*PPARA*	1.01	1.04	0.06	0.65	0.43	0.98
*ACOX1*	1.02	1.00	0.03	0.62	0.91	0.20
*HMGCS2*	0.89	0.91	0.05	0.74	<0.01	0.96
Hepatokines						
*FGF21*	-1.98	-1.75	0.58	0.77	<0.01	0.23
*ANGPTL4*	-0.25	-0.22	0.11	0.85	<0.01	0.68
Gluconeogenesis						
*PCK1*	0.99	1.15	0.04	0.01	<0.01	0.07
*PDK4*	-0.20	0.32	0.21	0.09	<0.01	0.52
*PC*	0.87	0.82	0.06	0.55	<0.01	0.16
Methionine cycle						
*BHMT*	1.14	1.04	0.11	0.52	<0.01	0.76
*MAT1A*	1.16	1.12	0.08	0.74	0.80	0.07

^1^ Data were log-transformed before statistics.

^2^ Largest standard error of the mean is shown.

^3^ Interaction of diet × time.

#### Oxidative stress

**Fig 5:**
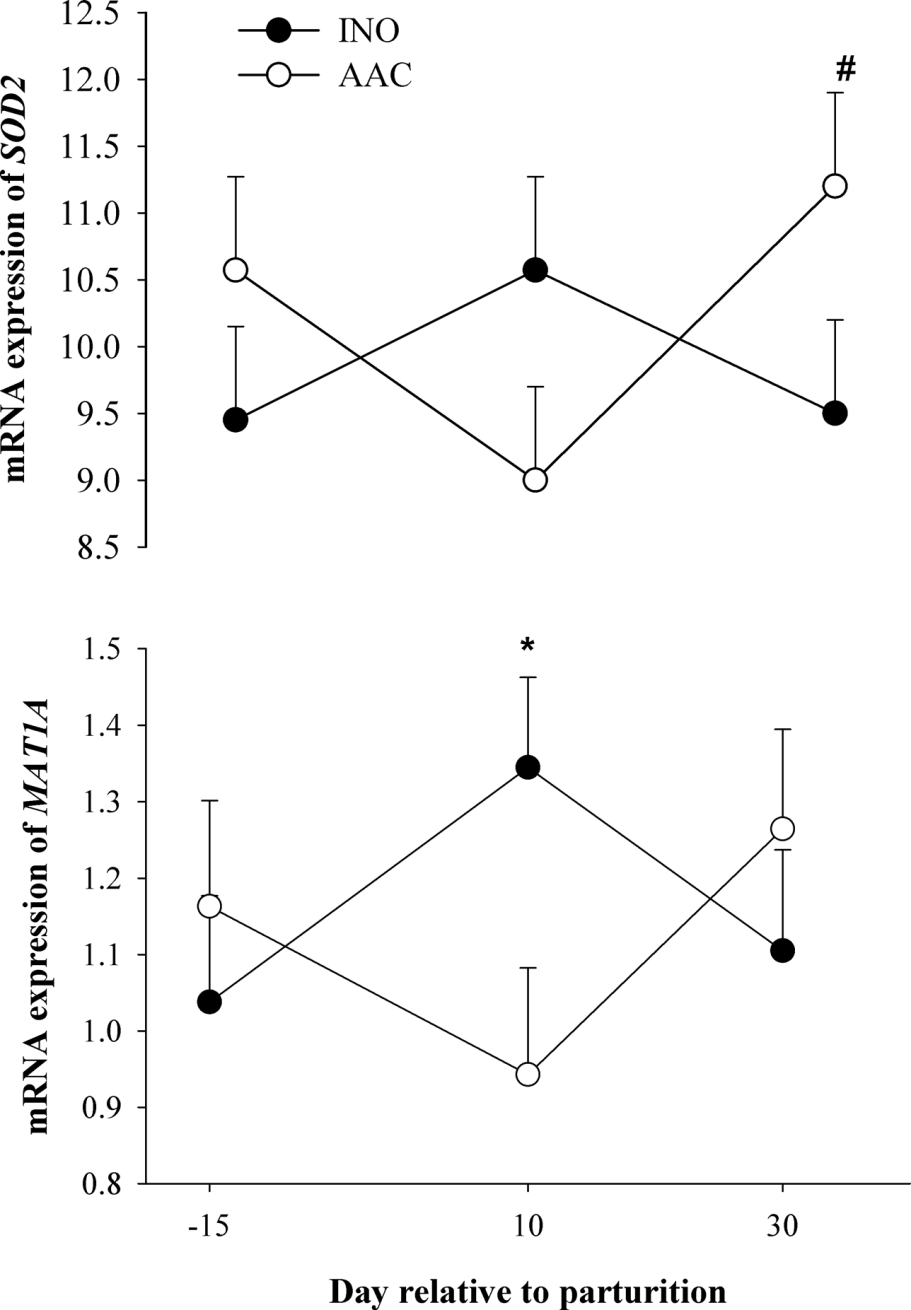
Effects of supplementing cows with inorganic (INO; n = 11) or organic (AAC; n = 9) trace minerals during the pre and postpartal periods on hepatic mRNA expression of genes related with oxidative stress (*SOD2*) and methionine cycle (*MAT1A*). Mean separations between diets at a given time point were evaluated when a D × T effect (P ≤ 0.10) was observed. Differences (*) were declared at P ≤ 0.05 and comparison between 10 and 30 d postpartum in AAC cows was declared (#) at P ≤ 0.05.

No overall effect of diet (*P* > 0.05) was detected on the expression of genes related with oxidative stress. Although there was a D × T effect (*P* < 0.04) for the expression of *SOD2*, differences between dietary treatments at each time point did not reach (*P* > 0.10) statistical significance. The interaction effect was due to the increase in *SOD2* expression from 10 to 30 d postpartum in cows fed AAC (*P* = 0.04) while in INO it remained unchanged (*P* = 0.18) (**Table 2**and **Fig 5**). The mRNA expression of *PON1* and *NOS2* decreased over time (*P* < 0.01) regardless of dietary treatment.

#### Metabolism and hepatokines

**Fig 6:**
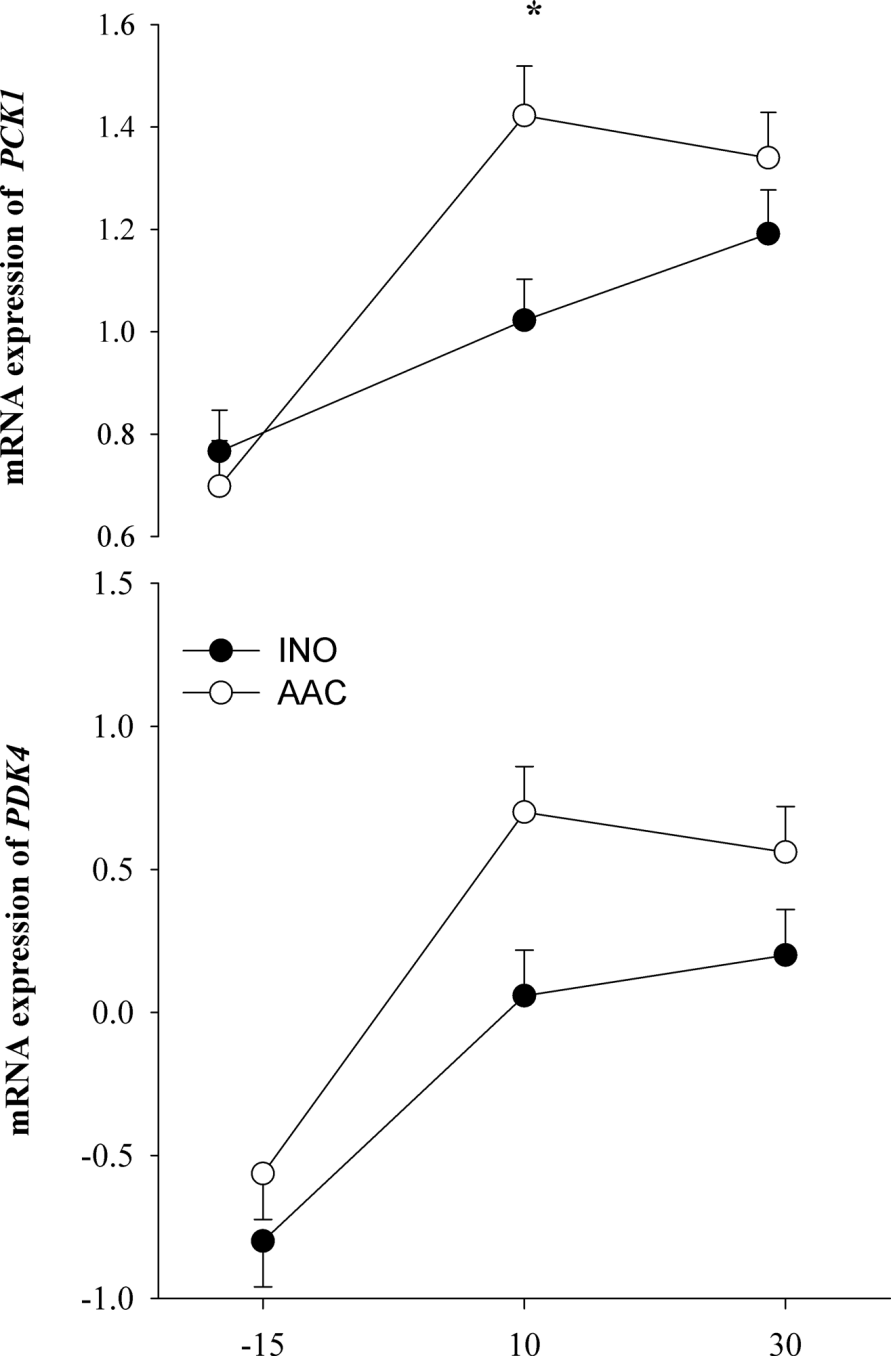
Effects of supplementing cows with inorganic (INO; n = 11) or organic (AAC; n = 9) trace minerals during the pre and postpartal periods on hepatic mRNA expression of genes related with gluconeogenesis. Mean separations between diets at a given time point were evaluated when a D × T effect (*P* ≤ 0.10) was observed. Differences (*) were declared at *P* ≤ 0.05 and trends (**) at *P* ≤ 0.10.

Although the expression of fatty acid metabolism-related genes (*PPARA*, *ACOX1*, and *HMGCS2*) and hepatokines (*FGF21* and *ANGPTL4*) was not affected by diet (*P* > 0.10), the expression of the gluconeogenic gene *PCK1* had a tendency (*P* = 0.07) for a D × T because its expression increased over time, primarily at 10 d (*P* < 0.01) postpartum, in AAC cows compared with INO (**Table 2**and **Fig 6**). There was an overall tendency (*P* = 0.09) for greater expression of *PDK4*, another gluconeogenic gene, in cows fed AAC compared with INO. The expression of the methionine cycle gene *MAT1A* had a tendency for a D × T (*P* = 0.07) because of a lower (*P* = 0.03) expression at d 10 in cows fed AAC compared with INO (**Table 2**and **Fig 5**). The expression of *HMGCS2* (ketogenesis), *FGF21* and *ANGPTL4* (hepatokines), *PC* (gluconeogenesis) and *BHMT* (methionine cycle) had a time effect (*P* < 0.01) due to greater expression at d 10.

## Discussion

During the transition period, dairy cows undergo a period of decreased liver function and increased inflammation and oxidative stress [1], which in turn might increase the requirement for trace minerals that are involved in several metabolic processes (i.e. enzyme structures, metabolic pathways, vitamin synthesis) and immune system integrity. Therefore, lack of these minerals could not only limit metabolic capacity but also immune system response, and supplementation through more available forms may overcome this. Production data and selected biomarkers of energy balance and liver cell damage were reported previously by Osorio et al. [13]. Briefly, supplementation with AAC improved milk production and milk protein yield at least in part due to an increase in postpartal DMI over time. Despite the lack of difference in concentration of plasma non-esterified (**NEFA**) and liver tissue triacylglycerol, the lower plasma concentration of γ-glutamyltransferase and total ketones indicated that the better performance in AAC cows was partly due to a reduction in liver damage and greater metabolic activity to handle NEFA [13]. The present study utilized a more comprehensive set of biomarkers of liver function, inflammation, and oxidative stress in plasma and liver tissue to better understand the extent to which AAC could alter these biological processes.

### Biomarkers of liver function

During an inflammatory response, pro-inflammatory cytokines induce immune stress and metabolic changes in liver that shift utilization of nutrients for the synthesis of unusual proteins in liver and cells of the immune system [21]. The major effect of cytokines on the liver is the stimulation of the acute-phase response, inducing positive APP synthesis and decreasing synthesis of negative APP [3]. The plasma concentrations of negative APP and total cholesterol during early lactation have been used previously to classify liver functionality into upper, upper-intermediate, lower-intermediate, and lower quartiles that reflect the state of liver function [1, 3]. Cows in the upper liver function quartile have higher cholesterol concentration than cows in the lower liver function quartile (5.2 vs. 3.6 mM/L) [1, 3]. Such response indicated a better liver function partly because of the association of total cholesterol with lipoprotein synthesis in liver and better DMI [1, 3]. In the current experiment, although blood cholesterol concentration over time increased in all cows, this increase and the response in DMI [13] were more pronounced for AAC than INO cows.

Plasma GOT and γ-glutamyltransferase increase when hepatic cells are damaged, hence, both are signs of a potential impairment in liver function. Cows in the upper compared with lower postpartal liver function quartile had lower GOT concentration (82.4 vs. 87.3 U/L) [3]. In humans, Zn supplementation attenuates apoptotic hepatocyte death and improves liver function in patients with liver disease [22]. Although in the present study the overall GOT concentration in AAC and INO were higher than those associated with optimal liver function during the transition period [3], the lower values detected in AAC cows are an additional sign that they had a better liver function.

### Biomarkers of inflammation

During the transition period, all dairy cows undergo some degree of inflammatory response that if uncontrolled could compromise milk production and reproductive performance [23]. Plasma concentration of albumin, a negative APP, often is decreased after calving [3], whereas concentrations of ceruloplasmin, SAA and haptoglobin (positive APP) increase after parturition [3, 23, 24]. Therefore, APP have been used to monitor both inflammation status during the peripartal period and potential alternatives to alleviate it.

Interleukin 6 is considered a pro-inflammatory cytokine that possesses a range of biological functions including activation of the immune response [25] and hepatic regulation of APP synthesis [26]. The latter is part of the response of the organism to disturbances in homeostasis. Although published data underscored a high-degree of animal variation, higher concentrations of this cytokine were associated with incidence of disorders post-partum [2]. The fact that blood leukocytes are most-sensitive to inflammatory stimuli that increase IL-6 and IL-1β around parturition indicates that these cytokines play a major role in the inflammatory response during the peripartal period [27].

Possible reasons for the reduction in albumin after calving include the impairment in hepatic function that can downregulate its synthesis [8], loss of serum proteins into the uterine lumen because of tissue remodelling [28], and channeling of amino acids towards the synthesis of mediators of inflammation [29]. Although excess intake of metals (e.g. Cu, Zn) has pro antioxidant effects [30], in non-ruminants, both Zn [31, 32] and Cu supplementation [33] have anti-inflammatory effects by reducing pro-inflammatory cytokines and edema. Therefore, it is not surprising that increasing availability of Zn is essential for an adequate response of the immune system [34].

Although the concentration of SAA tended to increase in the AAC group, the numerically lower concentration of ceruloplasmin in AAC cows indicated an inconsistent response of AAC on positive APP. The response in ceruloplasmin in AAC cows was associated with the decrease in liver Cu [13], which was in agreement with Bertoni et al. [35] who reported a positive correlation between liver Cu and ceruloplasmin. Ceruloplasmin is a Cu transport protein that oxidizes ferric iron (Fe^+3^) to ferrous iron (Fe^+2^) without the production of free Fe^+3^ which can cause oxidation and peroxidation in tissues [9]. However, the greater concentration of albumin after calving as well as the lower concentration of IL-6, haptoglobin and ceruloplasmin in AAC cows indicate that they experienced a lower inflammation status after parturition.

Although we did not observe a difference in tocopherol and β-carotene concentrations between treatments, the greater plasma retinol concentration in AAC cows could have been related with the lower inflammation status. A reduction of vitamin concentrations (e.g., tocopherol and retinol) in tissues (i.e., lung, liver, and heart) and plasma, at least in rodents, are associated with systemic inflammation [36]. Retinol concentration typically decreases around parturition due in part to the transfer of retinol and its derivatives into colostrum [37] and also because of the inflammation during this period [38]. Retinol is stored in the liver, and its transport to target tissues depends on retinol binding protein 4 (**RBP4**) [39]. As RBP4 is Zn-dependent [40] the decrease in plasma retinol during the transition period may be partly associated with a direct (diet) or indirect (immune response) [41] Zn deficiency. Although feeding AAC did not change the hepatic Zn concentration [13], the greater plasma retinol concentration in AAC cows could have contributed to the apparently lower inflammatory status [42, 43].

### Biomarkers of oxidative stress

The imbalance between reactive oxygen metabolite (**ROM**) production and the neutralizing capacity of antioxidant mechanisms leads to oxidative stress. Phases of high metabolic demand, e.g., transition period, enhance production of ROM, which consequently augment the requirement for antioxidants [44]. The latter can be produced endogenously by the organism (e.g. glutathione, taurine, bilirubin) and/or be derived from the diet [44, 45]. Oxidative stress plays a key role in the pathogenesis of diseases and has been identified as a link between nutrient metabolism and inflammation during the transition period [46]. Although there was no difference in concentration of ROM [13], nitric oxide is indirectly linked to ROM because it is a signaling molecule and when it is over-produced acts as a pro-inflammatory mediator that induces inflammation and also can injure cells [47].

Antioxidant activity can be evaluated via indices such as FRAP [17], where an increase in plasma values indicates a greater need for neutralizing ROM production, or via quantitating the oxygen-radical absorbing capacity (**ORAC**) of antioxidants in serum [48]. In the latter, the results denote the protection produced by antioxidants in plasma, with higher values indicating greater protection. Therefore, the lower concentration of nitric acid, its end products (NO_2_ and NO_3_), lower FRAP over time, and greater ORAC over time [13] in the AAC cows strongly indicate that the organic trace minerals were more efficacious in helping modulate the oxidative stress response. The fact that cellular antioxidant systems depend on the integrity of an enzymatic system that requires adequate intake of trace minerals such as Se, Cu, Zn, and Mn [34, 49] offers support to our conclusion.

### Genes related with the inflammatory response

The decrease in plasma albumin concentration is a reliable indicator of an inflammatory response and is closely related with liver function [3]. In line with this, the greater mRNA expression of *ALB* agrees with the greater plasma albumin concentration in cows fed AAC. The IL-10-mediated anti-inflammatory response represents an essential homeostatic mechanism that controls the degree and duration of inflammation [50]. During the anti-inflammatory response, IL-10 binds to the IL-10 receptor and activates the IL-10/JAK1/STAT3 cascade, leading to phosphorylated STAT3 homodimers translocating to the nucleus and activating the expression of target genes [50]. Although many cytokine receptors, including IL-6, generate their signals via the STAT3 pathway, the IL-10 receptor appears unique in promoting a potent anti-inflammatory response via STAT3 to antagonize pro-inflammatory signals that activate the innate immune response [51]. In this regard, the greater mRNA expression of *IL10* and *STAT3*, as well as the decrease of plasma IL-6 concentration in cows fed AAC, suggest that those cows were able to overcome the inflammation process during the transition period faster than cows fed INO. The mechanisms for how trace minerals affect the IL-10/JAK1/STAT3 pathway are not completely understood.

### Genes related with oxidative stress

Eukaryotic cells typically possess three evolutionarily-distinct forms of superoxide dismutases (SOD) that function to remove superoxide: a Cu-Zn containing SOD that localizes primarily to the cytosol (SOD1); a Mn containing SOD which resides in the mitochondrial matrix (SOD2); and a Cu-Zn containing SOD present in the extracellular space (SOD3) [52]. Between the two isoenzymes of SOD evaluated (*SOD1* and *SOD2*), *SOD2* expression was the only one with greater expression in AAC-supplemented cows, indicating that any effect of AAC supplementation was partly compartmentalized to the mitochondria. This organelle is the main site of ROM production in mammalian cells and its accumulation leads to energy depletion and ultimately cell death [53]. In wild-type mice on a normal chow diet, Mn supplementation increased SOD2 activity [54]. Therefore, the upregulation of *SOD2* with AAC is indicative of a potential increase in SOD2 activity and decreased mitochondrial oxidative stress. From the context of feeding INO, the response in *SOD2* expression also could be indicative that the Mn might not have been sufficient or effective in promoting the transcription of this gene. As such, there would have been a greater concentration of free superoxide radicals, thus, augmenting the likelihood of oxidative stress within liver.

Methionine adenosyltransferase, which is encoded by the gene *MAT1A*, is essential to catalyze the formation of S-adenosylmethionine (**SAM**), the principal biological methyl donor [55]. Synthesis of Cys and subsequently glutathione relies on SAM as a precursor [56]. Glutathione is the most-abundant endogenous antioxidant which scavenges free radicals to alleviate the risk of cellular damage [57]. An increase of SAM usually contributes to a decrease in hepatic oxidative stress through the up-regulation of glutathione synthesis. Therefore, the upregulation of *MAT1A* in INO cows at d 10 could partly be attributed to a greater need for antioxidants to scavenge ROM.

### Genes related with gluconeogenesis

Gluconeogenesis is central to the ability of high-producing dairy cows for maintaining an adequate glucose supply for the mammary gland [58]. The rate of total glucose increases markedly following parturition from 200–400 mmol/h to 700 mmol/h (3 kg/day) during peak lactation at a milk yield level of ~40 kg/d [59]. This tremendous increase of glucose needs is due to its use in the mammary gland for lactose, glycerol-3-phosphate, and NADPH synthesis [60], and also to support the immune response (e.g., increase of APP synthesis) [61].

Mitochondrial oxaloacetate (**OAA**) represents the entry point of most glucogenic substrates into gluconeogenesis pathway. Propionate is converted through mitochondrial propionyl-CoA carboxylase to OAA, whereas lactate and glucogenic AA are initially converted to pyruvate in the cytosol before being converted to OAA by mitochondrial pyruvate carboxylase (**PC**). The OAA can then be metabolized by phosphoenolpyruvate carboxykinase (**PCK**) to phosphoenolpyruvate and further to glucose or serve as an acetyl-CoA acceptor in the TCA cycle [62]. The presence of PCK activity in the cytosol (**PCK1**) and mitochondria (**PCK2**) further extends the possibilities to regulate the precursor entry into gluconeogenesis, and these enzymes are encoded by *PCK1* and *PCK2* [63]. Accordingly, it has been proposed that PCK1 is required for gluconeogenesis from amino acids and propionate, and PCK2 is more suited for gluconeogenesis from lactate [62]. Hence, the greater expression of *PCK1* in cows fed AAC indicates an increase in gluconeogenesis from AA and also from propionate. Although we did not measure metabolizable protein (**MP**) flow, because DMI and MP are positively correlated [14], the increase of DMI in the AAC treatment [13] partly supports the idea of greater post-absorptive AA and propionate availability for use in the gluconeogenic pathway. The lower metabolic costs required for the functioning of the immune system in AAC cows also would allow for greater availability of nutrients in those cows for other purposes [61, 64].

The greater expression of *PDK4* in cows fed AAC provides additional support for an overall increase in gluconeogenesis after calving in response to AAC. The PDK4 protein is located in the matrix of the mitochondria and when activated (e.g. low availability of glucogenic precursors) it inhibits the pyruvate dehydrogenase complex, hence, reducing the conversion of propionate to acetyl-CoA and increasing gluconeogenesis [65]. Therefore, although we did not detect differences in *PC* expression, the greater expression of both *PCK1* and *PDK4* in cows fed AAC, along with greater DMI, support the conclusion of an increase in overall gluconeogenesis. Furthermore, these data indicate that greater availability of glucose to the mammary gland is at least in part responsible for the greater milk yield in cows supplemented with AAC [13].

## Conclusions

The findings of this study reveal that supplementation with Zn, Mn, and Cu from AA complexes and Co from Co glucoheptonate during the peripartal period improved liver function as well as decreased inflammation and oxidative stress in transition cows. The better liver function with organic trace mineral supplementation enhanced gluconeogenesis partly through upregulating *PCK1*.

## Supporting Information

S1 File Description of protocols for RNA extraction, design and evaluation of primers, cDNA synthesis, and polymerase chain reaction (PCR). Table A. Features of primers used for qPCR analysis. GenBank accession number, hybridization position, sequence, and amplicon size of primers for *Bos taurus* used to analyze gene expression. Table B. Sequencing results of PCR products from primers of genes used for this experiment.
(DOC)
