# Supplementary material for: Immunometabolic Status during the Peripartum Period Is Enhanced with Supplemental Zn, Mn, and Cu from Amino Acid Complexes and Co from Co Glucoheptonate
Source: PLoS One. 2016 May 31;11(5):e0155804. doi: 10.1371/journal.pone.0155804 (PMC4887067; doi:10.1371/journal.pone.0155804)
Supplement: S1 File — Table A. Features of primers used for qPCR analysis. GenBank accession number, hybridization position, sequence, and amplicon size of primers for Bos taurus used to analyze gene expression. Table B. Sequencing results of PCR products from primers of genes used for this experiment. (DOC) [file pone.0155804.s001.doc]

**S1 FILE**

**RNA extraction**

Approximately 40 mg of frozen liver tissue was weighed and immediately placed in ice-cold 1 mL Qiazol reagent (Qiagen 75842; Qiagen Inc., Valencia, CA) for homogenization. After homogenization, the samples were centrifuged for 10 min at 12,000 × g at 4ºC to remove the insoluble material. The supernatant was transferred to a collection tube and incubated for 5 min on ice. Chloroform (200 µL) was added to each tube and the sample incubated at room temperature for 3 min. Subsequently, samples were centrifuged for 15 min at 12,000 × g at 4ºC, and the upper phase was transferred to a new collection tube without disturbing the mid and lower phases. A second wash was performed with 100% ethanol; 750 µL was added and transferred to a miRNeasy Mini Kit column (Cat. No: 217004, Qiagen). Genomic DNA was removed on column from RNA samples with RNase-free DNase I, using the recommended protocol provided with the miRNeasy Mini Kit. RNA concentration was measured with a NanoDrop ND-1000 spectrophotometer (Thermo Fischer Scientific; Wilmington, DE), while the RNA quality was assessed using the Agilent 2100 Bioanalyzer system (Agilent Technologies, Santa Clara, CA). The RNA samples with RNA integrity number ≥6.0 were considered of sufficient quality for analyses.

**Design and evaluation of primers**

Primers were designed using Primer Express (version 2.0 or 3.0) with min amplicon size of 80 bp (but when possible were preferred amplicon ≥ 100 bp) and limited 3’ G+C (Applied Biosystems, CA). Major part of the primers sets were designed to fall across exon–exon junctions. Primers were aligned against publicly available databases using BLASTN at NCBI and UCSC’s Cow (*Bos taurus*) Genome Browser Gateway (http://genome.ucsc.edu/cgi-bin/hgGateway).

Prior to qPCR the primers were tested in a 20 μL PCR reaction using the same protocol described for qPCR except the final dissociation protocol. For this purpose we used a universal reference cDNA (RNA mixture from 5 different tissues) to be sure to identify the gene. Five μL of the PCR product was run in a 2% agarose gel stained with ethidium bromide. The remaining 15 μL were cleaned using QIAquick® PCR Purification Kit (QIAGEN) and sequenced at the Core DNA Sequencing Facility of the Roy J. Carver Biotechnology Center at the University of Illinois, Urbana-Champaign. Only primers that presented a single band at the expected size and the right amplification product (verified by sequencing) were used for qPCR. The accuracy of a primer pair was also evaluated by the presence of a unique peak during the dissociation step at the end of qPCR.

**cDNA synthesis**

The quality of the extracted RNA samples was evaluated using the Agilent 2100 Bioanalyzer system (Agilent Technologies, Santa Clara, CA). The RNA samples with RNA integrity number >6.0 were considered of sufficient quality for analyses. The cDNA reaction was performed with 100 ng of RNA. The RNA was mixed with the Master Mix-I (MM1) containing 9 µL DNase/RNase free water and l µL random primers (Cat. no. 11; Roche, Mannheim, Germany). The mixture was incubated at 65°C for 5 min. After incubation, the MM1+RNA was kept on ice for at least for 3 min before adding 9 µL of Master Mix-II (MM2). The MM2 contained the following ingredients: 1.625 µL DNase/RNase free water, 4 µL 5X First-Strand Buffer (shipped with RevertAid enzyme (Fermentas), 1 µL Oligo dT18, 2 µL 10 mM dNTP mix (10 mM; Cat. No. 18427-088; Invitrogen), 0.25 µL of Revert aid (200 U/µL; Cat. No. EP 0442; Fermentas), and 0.125 µL of RNase inhibitor (40 U/μL; Cat. No. EO 0382; Fermentas). The final 20 µL vol (MM1+RNA and MM2) was incubated using the following temperature program: 25°C for 5 min, 42°C for 60 min and 70°C for 5 min. followed by 4°C.

**Polymerase Chain Reaction (PCR)**

The resulting cDNA was diluted (1:4) with DNase/RNase free water. After proper vortexing, a 4 µL diluted cDNA sample or standard curve dilution was carefully pipetted in triplicate into MicroAmp™ Optical 384-Well Reaction Plates. A 6 µL of SYBR Green Master Mix composed of the following ingredients was added to each well: 5 µL 1x SYBR Green (PerfeCTa SybrGreen), 0.4 µL of 10 µM forward primer, 0.4 µL of 10 µM reverse primer, and 0.2 µL DNase/RNase free water. The reactions was performed in an ABI Prism 7900 HT SDS instrument using the following conditions: 2 min at 50°C, 10 min at 95°C, 40 cycles of 15 s at 95°C and 1 min at 60°C. The specificity of the amplicons was verified with the dissociation protocol: 95°C for 15 s plus 65°C for 15 s. The data obtained were analyzed using the 7900 HT Sequence Detection Systems Software (version 2.2.1, Applied Biosystems).

**Table A.** Features of primers used for qPCR analysis. GenBank accession number, hybridization position, sequence, and amplicon size of primers for *Bos taurus* used to analyze gene expression.

| Accession # | Symbol | Primers^1^ | Primers (5’-3’) | bp^2^ |  |
| --- | --- | --- | --- | --- | --- |
| M73993.1 | *ALB* | F.1548 | AGTGCTGCACAGAGTCATTGGT | 80 | |
|  | *ALB* | R.1627 | GGCTTTGGGTACATATGTTTCATCA |  | |
| NM_001012671.2 | *STAT3* | F.3804 | GGTAGCATGTGGGATGGTCTCT | 110 | |
|  | *STAT3* | R.3913 | GCATCCCTAGAAACTCTGGTCAA |  | |
| NM_001076409.1 | *NFKB1* | F.172 | TTCAACCGGAGATGCCACTAC | 95 | |
|  | *NFKB1* | R.266 | ACACACGTAACGGAAACGAAATC |  | |
| NM_173966.3 | *TNF* | F. 174 | CCAGAGGGAAGAGCAGTCCC | 114 | |
|  | *TNF* | R. 287 | TCGGCTACAACGTGGGCTAC |  | |
| NM_001075260 | *SAA2* | F. 315 | AGCTCTTCACAGGCCTCATTC | 101 | |
|  | *SAA2* | R. 415 | CCACATGTCTTTAGCCCCTTC |  | |
| NM_001256556 | *CP* | F. 172 | GGTTGACACGGAACATTCCAA | 146 | |
|  | *CP* | R. 317 | GGCCTAAAAACCCTAACCAGACA |  | |
| NM_001040470.1 | *HP* | F.1210 | GGTTCGGAAAACCATCGCTA | 101 | |
|  | *HP* | R.1310 | CACTCGTGTCCCCTCCACTC |  | |
| NM_174093.1 | *IL1B* | F. 30 | ATTCTCTCCAGCCAACCTTCATT | 100 | |
|  | *IL1B* | R. 129 | TTCTCGTCACTGTAGTAAGCCATCA |  | |
| EU276074.1 | *IL10* | F.171 | GAAGGACCAACTGCACAGCTT | 98 | |
|  | *IL10* | R.268 | AAAACTGGATCATTTCCGACAAG |  | |
| NM_174615.2 | *SOD1* | F. 256 | TGGAGATGCACAGATACACAGCTA | 101 | |
|  | *SOD1* | R. 356 | GCTGTCACATTGCCCAGGT |  | |
| NM_201527.2 | *SOD2* | F.620 | TGTGGGAGCATGCTTATTACCTT | 95 | |
|  | *SOD2* | R.714 | GCATCCCTAGAAACTCTGGTCAA |  | |
| BC112599.1 | *PON1* | F. 1088 | ATCCGTACCCCTACCCTGACA | 100 | |
|  | *PON1* | R. 1188 | GGGTCAGCGTTCACTGTTGA |  | |
| NM_001076799.1 | *NOS2* | F. 3283 | CTGAAGCAGCTGATGGCTACTG | 89 | |
|  | *NOS2* | R. 3372 | ATGATAGCGCTTCTGGTTCTTGAG |  | |
| NM_001034036.1 | *PPARA* | F.729 | CATAACGCGATTCGTTTTGGA | 102 | |
|  | *PPARA* | R.830 | CGCGGTTTCGGAATCTTCT |  | |
| NM_001035289.2 | *ACOX1* | F.180 | ACCCAGACTTCCAGCATGAGA | 100 | |
|  | *ACOX1* | R.279 | TTCCTCATCTTCTGCACCATGA |  | |
| NM_001045883.1 | *HMGCS2* | F.837 | TTACGGGCCCTGGACAAAT | 100 | |
|  | *HMGCS2* | R.936 | GCACATCATCGAGAGTGAAAGG |  | |
| XM_002695200.2 | *FGF21* | F.223 | CAGAGCCCCGAAAGTCTCTTG | 100 | |
|  | *FGF21* | R.348 | AAAGTGCAGCGATCCGTACAG |  | |
| NM_001046043.2 | *ANGPTL4* | F.28 | AGGAAGAGGCTGCCCAAGAT | 109 | |
|  | *ANGPTL4* | R.136 | CCCTCTCTCCCTCTTCAAACAG |  | |
| JQ733520.1 | *PCK1* | F. 601 | AAGATTGGCATCGAGCTGACA | 120 | |
|  | *PCK1* | R. 720 | GTGGAGGCACTTGACGAACTC |  | |
| NM_001101883 | *PDK4* | F. 2484 | ACAAATAGCCTGCGGGTAAGAG | 102 | |
|  | *PDK4* | R. 2585 | CGTACGCTTTACGGCTTTCAT |  | |
| NM_177946 | *PC* | F. 3577 | GCAAGGTCCACGTGACTAAGG | 80 | |
|  | *PC* | R. 3700 | GGCAGCACAGTGTCCTGAAG |  | |
| NM_001011679.1 | *BHMT* | F. 302 | GCTCTCCTCGTCCATCCTCAT | 102 | |
|  | *BHMT* | R. 404 | CCGTTCTAGGATGCCCTTCTT |  | |
| NM_001046497 | *MAT1A* | F. 32 | CAGATTCCAGATTCCAGT | 110 | |
|  | *MAT1A* | R. 142 | CAAGGGCTTTGACTTTAAG |  | |

^1^ Primer direction (F – forward; R – reverse) and hybridization position on the sequence.

^2^ Amplicon size in base pair (bp).

**Table B.** Sequencing results of PCR products from primers of genes used for this experiment.

| Gene | Sequence |
| --- | --- |
| *ALB* | GCGATCAGTTTCTTCGTCTCGTACACCTGATGAACATATGTACCCAAAGCCAC  ACAATGACTCTGGGCAGCACTAACC |
| *STAT3* | GCATCCCTCTACGAGCACGGCTAGATGTGGTCGGCTACAGCCATCTTGTCTCAGTTGACCAGAGTTTCTAGGGATGCAA |
| *NFKB1* | CGATATCTTCGTGTCAAGCAAAAGTATTCGCAACACTGGAAGCACGAATGACAGATGCCTGTATACGGGGCATCAGAAGGCCGTA |
| *TNF* | TCACTCTCCGGGGCAGCTCCGGTGGTGGGACTCGTATGCCAATGCCCTCAT GGAA |
| *PC* | GATCATAGGAGTACAGAACTCATCTGGAAGAATCGAGTGACCACGCTGAGACTGGCAGCCTGACCATCCCCGACCCCTGCCTTCAGGACACTGTGCTGCCAGA |
| *HP* | CACTAGCCAGATCGAGAACAGGTGAAGCAAGCTGGCATCGAATCGGCCTTTCACTCTCGATGAATGTGCAAAACCC |
| *IL1B* | ACAGCCATGGCACCGTACCTGAACCCATCAACGAAATGATCGGCTTACGTCACAGTGGACAGAGCACAATAGCACCCCC |
| *IL10* | GCACGAGTACTCTCGTCACTAGGAGTACCTTTAAGGGTTACCTGGGTGTGCCAAGGCCTTGTCCGGAAATGATCCAGTTTACGA |
| *SOD1* | GTCCAAAAACCGGTGGGCCAAAAGATGAAGAGAGGCATGTTGGAGACCTGGGCAATGTGACAGCT |
| *SOD2* | GCATGTTTGGCCGATTATCTGAGGCCATTTTGGAATGTGATCAACTGGGAGAATGTAACTGCAATAC |
| *PON1* | GATAACTCATATCACGTCTGTCCACATCTGTGGGGGCCACAGTGATCTCATCTCAACAGTGAACGCTGACCCAA |
| *NOS2* | ACGAGGAAACGGGTGGAGGACTATTTCTTCCAGCTCAAGAACCAGAAGCGCTATCATG |
| *PPARA* | CGAGATCTGAAGCAAATTGAGGCAGAAATCCTTACGTGTGAGCATGACCTAGAAGATTCCGAAACCGCGA |
| *ACOX1* | ATCCTCGTATCCGCGTTCAGGGTGCGTTTAAGAAGAGTGCCATCATGGTGCAGAAGATGAGGAAATCCCC |
| *HMGCS2* | CACTAGCCAGATCGAGAACAGGTGAAGCAAGCTGGCATCGAATCGGCCTTTCACTCTCGATGAATGTGCAAAACCC |
| *FGF21* | GACGTAGCAGGCGCTATTCAGATCTTGGGAGTTAAACATCCAGGTTTCTCTGCCAGGGGCCAGATGGGAAGCTGTACGGATCGCTGCACTTTTA |
| *ANGPTL4* | GCCCATCAGCATCCTCAACCGTGAAGCGGCCAGTATTTCCACTCCATTTCCAAGGGAAGA |
| *PCK1* | GCCATGTGTACAGCAGTCGCATCATGACGAGGATGGGCACCAGCGTCCTGGAAGCGCTGGGGGACGGCGAGTTCGTCAAGTGCCTCCACAAA |
| *PDK4* | CTCGTGGATGGGCTCTATTTCTAATTGTTGTGAATGAGAGCTAATGCATGAAAGCCGCTAAAGCGTACGA |
